# Mediating effect analysis of professional identity between emotional intelligence and work readiness of newly graduated nursing students

**DOI:** 10.21203/rs.3.rs-6353643/v1

**Published:** 2025-06-10

**Authors:** Liping Chen, Liping Wu, Heping Liao, Qin Lin, Yetao Luo

**Affiliations:** Department of Endocrinology, Chongqing Medical University Affiliated Children’s Hospital; Department of Nursing, Chongqing Medical University Affiliated Children’s Hospital; School of Nursing, Chongqing Medical University; Department of Endocrinology, Chongqing Medical University Affiliated Children’s Hospital; Department of Nosocomial Infection Control, Second Affiliated Hospital, Army Medical University

**Keywords:** Newly Graduated Nursing Students, Professional Identity, Job Readiness, Emotional Intelligence, Nursing Education

## Abstract

**Aim:**

To collect relevant data through a cross-sectional survey study and analyze the mediating effect of professional identity in the relationship between emotional intelligence and work readiness.

**Background:**

Poor work readiness makes new nursing grads leave the profession. Emotional intelligence and professional identity impact it, so it’s important to clarify the relationship between them to enhance the work readiness of nursing students.

**Methods:**

Cluster sampling was performed on newly graduated nursing students from Chongqing, China. Data were collected using the Emotional Intelligence Scale, Nurses’ Work Readiness Scale, and Nurses’ Professional Identity Scale. The SAS software (version 9.4) was used for data processing and analysis. Independent sample t-tests and one-way ANOVA were performed to determine influencing factors. Amos (version 29.0) was used to model mediation effects. The maximum likelihood estimation method was used to estimate the model parameters, with adjustments made based on the correction index.

**Results:**

In total, 453 valid questionnaires were obtained. The total scores for professional identity, emotional intelligence, and work readiness of the participants were 66.79 ± 11.35, 90.09 ± 14.09, and 271.10 ± 44.26, respectively. professional identity correlated with the other two, mediating their relation, accounting for 69.99% of total effects.

**Conclusions:**

Professional identity is an important mediator between emotional intelligence and work readiness. School teachers and healthcare professionals should prioritize developing nursing students’ emotional intelligence and professional identity during education. Enhancing professional identity, fostering a sense of professional honor, and improving work readiness can reduce turnover rates and stabilize the nursing workforce.

**Implications for Nursing and/or Health Policy:**

Both professional identity and emotional intelligence are positively correlated with work readiness, which deserve more attention. Nursing educators and mangers should cultivate professional identity early.

## 1. Background

With evolving nursing concepts and service models for disease treatment, the role of nurses in patient health management has become increasingly prominent. The Chinese government has issued the China Nursing Career Development Plan (2021-2025), emphasizing improvements in mobilizing the nursing workforce and increasing the number of nurses(NHSC, 2022). However, recent statistics reveal that while China has 5.637 million registered nurses, the nurse-to-population ratio is only four per 1,000 people, below the World Health Organization’s minimum standard and significantly lower than ratios in developed countries (WHO,2023; Chen et al.,2024). Newly graduated nursing students, defined as nurses who have recently graduated from school and are entering clinical practice, represent a vital component of the future nursing workforce (Zhu et al.,2023). Numerous studies (See et al.,2023; Kaldal et al.,2023; Alharbi et al.,2023; Hu et al.,2023) indicate that due to the high risk, intensity, and standards of clinical nursing work, newly graduated nursing students face a series of adaptive challenges from school campuses to clinics, leading to work frustration and a high turnover rate, with approximately 75% of new nurses expressing a strong desire to leave within their first year of employment, and 30–60% resigning during this period. Therefore, facilitating a smoother transition for new graduates in the early stages of their career development is necessary to improve clinical care quality and stabilize the nursing workforce.

Work readiness measures an individual’s degree of readiness in terms of knowledge, skills, and attitudes before entering the workplace. Among newly graduated nursing students, work readiness is positively correlated with job satisfaction and work engagement, serving as the basis for a good transition from student to professional nurses (Walker et al.,2013). However, evidence suggests that nursing students often lack adequate preparation for clinical decision-making, communication, coordination, and emergency handling (Ogawa et al.,2024; Novalia et al.,2021; Najafi et al., 2023; Horii et al.,2021). According to one study, only 10% of hospital administrators in China consider newly graduated nursing students work-ready, while 63% of clinical tutors believe they require additional support (He et al., 2021). In-depth understanding of factors influencing work readiness could guide nursing schools in enhancing clinical orientation and optimizing training programs. Emotional intelligence is the ability to recognize, understand, express, and manage one’s own and others’ emotions (Torre et al.,2023). It significantly impacts newly graduated nurses’ ability to handle work stress, build nurse-patient relationships, and foster teamwork. Emotional intelligence is positively correlated with nurses’ clinical communication skills, job satisfaction, professional commitment, and patient satisfaction. It is an important skill for managing conflict, stress, negative events, and workplace violence (Giménez-Espert et al., 2018). However, a study by Zhang et al. involving multiple provinces and cities in China found that only 15% of clinical nurses exhibited high emotional intelligence (Zhang et al.,2024; Zhang et al.,2024). Similarly, research by Deng Huaxia et al. revealed that nurses with ≤ 5 years of experience often have emotional intelligence that needs further improvement (Deng et al.,2024). Professional identity reflects an individual’s recognition and positive evaluation of their status and responsibilities (Li et al.,2024). Its formation is influenced by positive evaluation of society, personal interest, ambition, and material rewards. Well-formed professional identity is both an intrinsic motivating factor for personal development and the key to overcoming professional externalities and unifying personal and professional values. Surveys (Li et al.,2018; Chen et al.,2022; Zhang et al.,2017) indicate that 74.4% of nurses with ≤ 1 year of experience express a willingness to leave their job in the first year, with low professional identity being a key factor in their decision. Strengthening professional identity is therefore an urgent problem in nursing education and management.

The analysis above highlights an intrinsic causal link between work readiness, emotional intelligence, and professional identity, all of which significantly impact career stability among newly graduated nurses. While good work readiness is necessary for a smooth career start, emotional intelligence and professional identity also play critical roles. Understanding the interplay and contribution of these factors can inform strategies to enhance nursing education and workforce management, ultimately improving job retention among new graduates. However, to the best of our knowledge, there is limited research on how emotional intelligence and professional identity affect work readiness. Therefore, this study analyzed the relationship between work readiness, emotional intelligence, and professional identity and used mediation analysis to identify the key factors and their relative contributions.

## 2. Methods

### 2.1 Research design

This study employed a cross-sectional design.

### 2.2 Participants

The participants were newly graduated nursing students in Chongqing, China. The sample size was estimated using Kendall’s sample size estimation method (Fang,2007), which recommends 5–10 times the number of questionnaire items, with a 20% allowance for attrition. With 70 questionnaire items, the sample size was calculated as: number of questionnaire items (70) × (5–10) × (1 + 20%) = 420–840. The inclusion criteria were as follows: (a) Completion of all required nursing courses; (b) newly graduated nursing students; (c) normal cognitive and comprehension abilities; and (d) informed consent to participate.

### 2.3 Questionnaire

The general information questionnaire collected data on age, gender, education background, place of residence, voluntary selection of the nursing major, and confidence in nursing work.

Emotional Intelligence Scale: Developed by Wong et al. and translated into Chinese by Wang (Wang, 2010). It measures four dimensions: emotional self-assessment (four items), others’ emotional assessment (four items), emotional application (four items), and emotional control (four items). The scale was scored on a 7-point Likert scale, ranging from 1 (strongly disagree) to 7 (strongly agree), with higher scores indicating higher emotional intelligence.

Professional Identity Scale: Compiled by Liu et al. (Liu et al., 2011), it includes five dimensions: professional self-concept (six items), benefits of staying and risks of leaving the job (four items), social comparison and self-reflection (two items), autonomy of career choice (two items), and social persuasion (three items, including one reverse-scored item). The scale is scored on a 5-point Likert scale, ranging from 1 (very inconsistent) to 5 (very consistent), with higher scores indicating stronger professional identity.

Work Readiness Scale: Compiled by Walker and translated into Chinese by Li Jiaying et al. (Li et al.,2020). It includes five dimensions: job competitiveness (eight items), social skills (nine items), career development (nine items), organizational acumen (seven items), and personal work characteristics (four items). The scale is scored on a 10-point Likert scale, ranging from 1 (completely disagree) to 10 (completely agree), with items 34–37 being reverse-scored. Higher scores indicated better work readiness.

### 2.4 Data collection

The questionnaire was electronically generated via Wenjuanxin(www.wjx.cn) in November 2023. A staff member who managed new graduate nursing students in the researched schools was contacted to explain the purpose of the study, obtain their consent, and send the link to the electronic questionnaire via WeChat or QQ to be completed by new graduate nursing students who met the inclusion criteria.

### 2.5 Bias control

To minimize bias, the questionnaire contained standardized instructions explaining the purpose and content of the study and was anonymous. Participants could only submit the form after answering all questions, and duplicate submissions from the same IP were not considered. Data with filling time < 400s, contradictory answers, or uniform answers were excluded following manual review by two researchers.

### 2.6 Data analysis

The SAS software (version 9.4) was used for data sorting and analysis. Normally distributed quantitative data were described using mean ± standard deviation, with group comparisons analyzed via independent sample t-tests and variance analysis. Pairwise comparisons were performed using the SNK-q test. Categorical data were described as case number and rate. Pearson’s correlation analysis was performed to analyze the correlations between the scales. Variables with a single factor (P < 0.05) were included in the multivariate linear regression model, screened using a stepwise method (inclusion criteria: P < 0.05, exclusion criteria: P ≥ 0.05). The mediation effect model was established using AMOS (version 29.0); the model parameters were estimated using the maximum likelihood estimation method. The model was adjusted based on the correction index. The 95% confidence interval for effects was calculated by repeated sampling 5000 times. A bilateral P < 0.05 was considered statistically significant.

## 3. Results

Four hundred ninety-six newly graduated nursing students completed a questionnaire. After excluding 10 questionnaires with answer times < 400s, 14 with identical answers for all entries, and 12 with logically contradictory options, 453 valid questionnaires were obtained, resulting in a valid recovery rate of 91.33%. Table 2 shows the general characteristics of the participants. The professional identity, emotional intelligence, and work readiness scores of the newly graduated nursing students were 66.79 ± 11.35, 90.09 ± 14.09 and 271.10 ± 44.26 points, respectively (Table 1).

### 3.1 Correlation between professional identity, emotional intelligence, and work readiness

Statistically significant correlations were observed between work readiness, professional identity, and emotional intelligence among the participants (P < 0.05, Table 2).

### 3.2 Factors influencing career identity, emotional intelligence, and work readiness

Univariate analysis showed that multiple factors influenced participants’ professional identity, emotional intelligence, and work readiness scores (Table 3). Variables with P < 0.05 in the single factor were included in the multifactor linear regression model, which was refined using the stepwise method (inclusion criteria: P < 0.05, exclusion criteria: P ≥ 0.05). Table 4 shows the variable assignment table. Independent factors affecting work readiness included age, sex, frequency of online theory courses since the COVID-19 pandemic, professional identity, and emotional intelligence (Table 5).

### 3.3 Analysis of intermediation effects

**Figure 1:**
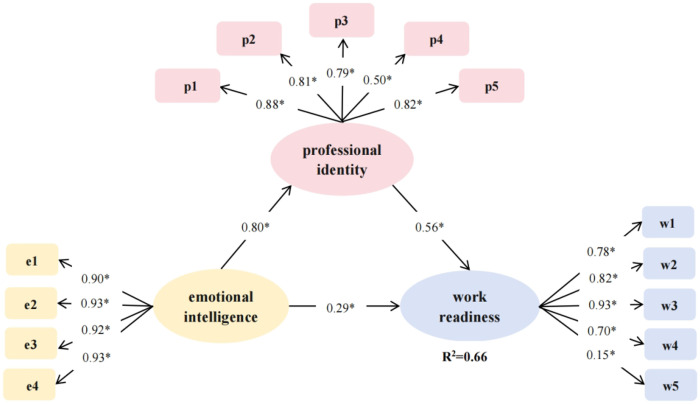
Mediation effect model of new graduate nursing students’ work readiness with professional identity and emotional intelligence (*P<0.01)

Figure 1 includes the mediation effect model for work readiness, professional identity, and emotional intelligence. Model fit indices indicated acceptable fit: χ2/df ratio = 238.064/66 = 3.607, GFI = 0.934, CFI = 0.970, IFI = 0.971, RMSEA = 0.076. Emotional intelligence and professional identity had direct effects on work readiness, with effect sizes of 0.289 (95% CI, 0.106–0.457) and 0.562 (95% CI, 0.395–0.729), respectively. Additionally, emotional intelligence indirectly influenced work readiness through professional identity, with a mediating effect of 0.452 (95% CI = 0.319–0.603), accounting for 60.99% of the total effect. Together, professional identity and emotional intelligence explained 66.00% of the variance in work readiness (Table 6).

## 4. Discussion

This study analyzed the role positioning and quantitative relationship between emotional intelligence and professional identity in shaping work readiness. Results showed that professional identity and emotional intelligence both positively predicted work readiness, while professional identity mediated the relationship between emotional intelligence and work readiness, accounting for 60.99% of the total effect. This shows that professional identity not only independently affects work readiness but also plays an important mediating role between emotional intelligence and work readiness. Jiang et al. (Jiang et al.,2024; Levin et al., 2022; Pirzadeh et al., 2023) similarly found that professional identity greatly affects work readiness and performance, reaffirming its central role. These findings provide a new perspective for educational institutions and healthcare organizations to improve nursing students’ work readiness and adaptability.

This study revealed that the work readiness score of newly graduated nursing students in Chongqing (271.10 ± 44.26) was higher than that of intern nursing students in Shandong, China (264.91 ± 59.97), newly employed nurses in Yunnan (259.31 ± 48.58), and newly graduated nursing students in Guangzhou (262.20 ± 44.97), but lower than higher vocational nursing students in Henan (273.09 ± 58.58). This may be due to differences in regional culture, research populations, and economic development levels (Xing et al., 2024; Song et al., 2023; Chen et al., 2022; Pan et al., 2023). Additionally, junior college nursing students scored higher than undergraduate nursing students, likely because higher-vocational colleges emphasize skill-based training over the theoretical and scientific research focus of undergraduate programs. This study also analyzed the relationship between nursing students’ gender, family support, voluntary career choices, cadre experience, and scholarships in relation to work readiness. Male nursing students displayed significantly higher work readiness than females, potentially due to the demand for male nurses in operating rooms, orthopedics, intensive care medicine, and emergency departments. Gender-based hiring preferences may affect job readiness. Nursing students with family support and those who voluntarily chose nursing exhibited higher work readiness scores, aligning with Chen et al.’s findings (Chen et al., 2022). These students demonstrated greater enthusiasm for learning and stronger nursing knowledge and skills. Cadre experience during their school years was associated with higher work readiness. Work experience can effectively train students to communicate with others, solve problems, and coordinate relationships. These abilities correspond to social skills and organizational acumen in work readiness, making them more confident in coping with clinical nursing work. Scholarship recipients also scored higher, reflecting their superior academic-related comprehensive qualities and adaptability skills, which support a smoother transition into professional roles.

This study identified a positive correlation between emotional intelligence and work readiness (r = 0.696, P < 0.001), indicating that students with higher emotional intelligence are better equipped to evaluate and manage emotions, use emotions reasonably, and improve work readiness. Emotional intelligence serves as an important psychological motivation resource, promoting positive work performance (Zhang et al., 2021). Nursing students with high emotional intelligence tend to have a strong sense of psychological control; are good at capturing the emotions of patients, colleagues, and leaders; manage and regulate their own emotions; change their thinking in time when facing conflicts; and adopt coordinated conflict management strategies to maintain good emotional relationships. In this study, the average emotional intelligence score was 90.09 ± 14.09, which was considered medium. Studies have shown that emotional intelligence can be improved through training. Future nursing educators should integrate emotional intelligence training into nursing humanities courses and professional training. Strategies such as mindfulness courses, academic lectures, special training, and social practice can help nursing students improve emotional management skills, thereby enhancing emotional intelligence and work readiness (Zeng et al., 2024). A positive correlation was also observed between professional identity and work readiness (r = 0.680, P < 0.001), consistent with Xu Duanya et al.’s findings (Xu et al.,2023). Professional identity, as a work-related value, significantly influences students’ commitment to nursing. A strong professional identity fosters greater enthusiasm and dedication, serving as a key indicator of good work readiness.

## 5. Limitations

The research participants were exclusively from Chongqing, an important city in western China, with no samples collected from other regions. This geographic limitation may have introduced sampling bias, potentially affecting the result’s accuracy. Additionally, the cross-sectional study design could not establish a causal relationship among the variables. Future studies should employ longitudinal or experimental designs to validate the causal hypotheses proposed.

## 6. Conclusion

This study found that emotional intelligence and professional identity significantly influence job readiness, with professional identity serving as a key mediator between emotional intelligence and job readiness. These findings underscore the need for schools, medical institutions, and healthcare management institutions to prioritize the development of nursing students’ emotional intelligence and professional identity. Efforts should focus on strengthening professional identity and fostering a sense of professional honor, improving nurses’ social and economic status, improving their job readiness, and reducing turnover rate.

## 7. Implications for Nursing & Health Policy

Our findings have several practical implications. First, the work readiness, professional identity, and emotional intelligence of new nurses need further improvement. Both professional identity and emotional intelligence are positively correlated with work readiness, which deserve more attention from nursing teachers and nursing managers. Second, professional identity plays a mediating role between emotional intelligence and work readiness. Nursing educators should pay attention to and cultivate the professional identity of nursing students in the early stage of their enrollment.

## Figures and Tables

**Table 1:** Scores of work readiness, professional identity, and emotional intelligence of newly graduated nursing students

Scale	Score	Score of item	Cronbach’s coefficient
**work readiness**			
job competitiveness(w1)	54.84 ± 11.68	6.85 ± 1.46	0.944
social skills(w2)	63.77 ± 13.88	7.09 ± 1.54	0.944
career development(w3)	69.76 ± 12.9	7.75 ± 1.43	0.933
organizational acumen(w4)	60 ± 9.41	8.57 ± 1.34	0.947
personal work characteristics(w5)	22.74 ± 7.79	5.68 ± 1.95	0.849
Total	271.1 ± 44.26	7.33 ± 1.20	0.818
**professional identity**			
professional self-concept(p1)	23.41 ± 4.84	3.9 ± 0.81	0.933
benefits of staying on the job and risks of leaving the job(p2)	14.96 ± 3.27	3.74 ± 0.82	0.855
social comparison and self-reflection(p3)	12.32 ± 1.94	4.11 ± 0.65	0.693
autonomy of career choice(p4)	7.69 ± 1.58	3.84 ± 0.79	0.188
social persuasion(p5)	8.42 ± 1.62	4.21 ± 0.81	0.890
Total	66.79 ± 11.35	3.93 ± 0.67	0.874
**emotional intelligence**			
self-emotional assessment(e1)	22.68 ± 3.79	5.67 ± 0.95	0.890
other people’s emotional assessment(e2)	22.63 ± 3.59	5.66 ± 0.9	0.876
emotional application (e3)	22.31 ± 3.74	5.58 ± 0.94	0.840
emotional control (e4)	22.48 ± 3.88	5.62 ± 0.97	0.881
Total	90.09 ± 14.09	5.63 ± 0.88	0.955

**Table 2:** Correlation analysis of work readiness, professional identity, and emotional intelligence of new graduate nursing students

Scale	Work readiness	Professional identity	Emotional intelligence
**Work readiness**	1	-	-
**Professional identity**	0.680^a^	1	-
**Emotional intelligence**	0.696^a^	0.733^a^	1

Note:

a P < 0.001

**Table 3:** Univariate analysis of professional identity, emotional intelligence and work readiness of new graduate nursing students

Category	Emotional intelligence	t/F	P	Professional identity	t/F	P	Work readiness	t/F	P
Age									
≤ 21(a)	91.91 ± 13.96	2.550	0.055	68.11 ± 11.68b	4.712	0.003	266.75 ± 49.99d	4.013	0.008
22(b)	88.54 ± 12.23			64.28 ± 10ad			264.22 ± 37.56d		
23(c)	88.4 ± 15.66			66.51 ± 11.41			274.59 ± 41.14		
≥ 24(d)	92.41 ± 14.66			69.52 ± 12.2b			283.01 ± 48.05ab		
Gender									
male	94.83 ± 12.84	2.233	0.026	70.1 ± 11.59	1.935	0.054	298.33 ± 42.5	4.147	< 0.001
female	89.64 ± 14.14			66.47 ± 11.29			268.46 ± 43.58		
Residency									
urban	90.03 ± 13.54	−0.092	0.927	66.12 ± 11.62	−1.271	0.204	269.35 ± 42.82	−0.850	0.396
rural	90.16 ± 14.66			67.48 ± 11.06			272.88 ± 45.71		
Only child									
yes	91.15 ± 13.54	0.981	0.327	66.32 ± 12.16	−0.540	0.589	276.27 ± 48.2	1.531	0.127
no	89.7 ± 14.3			66.97 ± 11.05			269.15 ± 42.59		
Educational background									
junior college or below	92.49 ± 15.27	3.238	0.001	70.19 ± 11.22	5.948	< 0.001	276.03 ± 48.34	2.106	0.036
bachelor degree or above	88.15 ± 12.76			64.04 ± 10.72			267.09 ± 40.3		
Whether any immediate family members are nurses									
yes	94.03 ± 15.76	1.696	0.091	69.47 ± 10.96	1.432	0.153	278.09 ± 53.18	0.958	0.339
no	89.78 ± 13.92			66.58 ± 11.37			270.53 ± 43.48		
Whether your parents support you becoming a nurse									
yes	90.89 ± 13.81	3.881	< 0.001	68.07 ± 10.66	8.139	< 0.001	272.75 ± 44.65	2.542	0.011
no	82.07 ± 14.54			53.93 ± 10.08			254.44 ± 36.61		
Whether you choose the nursing profession voluntarily									
yes	92.14 ± 12.86	6.768	< 0.001	69.02 ± 9.87	10.957	< 0.001	276.55 ± 41.82	6.400	< 0.001
no	78.93 ± 15.37			54.63 ± 11.28			241.26 ± 45.63		
Have you ever served as a student leader									
yes	91.21 ± 13.56	2.323	0.021	67.86 ± 11.24	2.763	0.006	276.66 ± 43.11	3.724	< 0.001
no	87.99 ± 14.86			64.78 ± 11.34			260.61 ± 44.63		
Have you ever received a scholarship									
yes	92.24 ± 13.28	2.810	0.005	68.2 ± 11.01	2.279	0.023	278.04 ± 43.39	2.901	0.004
no	88.5 ± 14.49			65.75 ± 11.51			265.94 ± 44.27		
Frequency of online theory courses since the COVID-19									
≤ 5 times/week(a)	89.99 ± 15.86	0.472	0.624	67.37 ± 11.75	0.592	0.554	271.02 ± 49.4	3.152	0.044
6–10 times/week(b)	89.47 ± 13.04			66.07 ± 10.57			265.6 ± 41.35c		
≥ 11 times/week(c)	91.03 ± 13.4			67.1 ± 11.92			278.31 ± 41.21b		
Ways of teaching practical skills courses since the COVID-19									
predominantly offline teaching	90.84 ± 13.59	1.513	0.131	67.34 ± 10.58	1.300	0.195	272.8 ± 45.55	1.090	0.276
Predominantly online teaching	88.76 ± 14.89			65.82 ± 12.6			268.07 ± 41.82		
Modes of participation in internships									
Mainly clinical placements	90.15 ± 14.09	0.775	0.439	66.85 ± 11.39	0.896	0.371	271.27 ± 44.28	0.720	0.472
Primarily online internships	85.67 ± 15			62.67 ± 7.87			258.17 ± 44.31		
Whether your willingness to engage in nursing has increased during the COVID-19 pandemic									
yes	93.92 ± 12.06	8.191	< 0.001	71.59 ± 8.72	15.629	< 0.001	281.89 ± 42.01	7.725	< 0.001
no	82.59 ± 14.79			57.38 ± 9.96			249.93 ± 40.9		
Are you confident in clinical nursing practice									
yes	93.97 ± 11.92	10.194	< 0.001	70.86 ± 8.79	15.940	< 0.001	282.16 ± 40.19	9.805	< 0.001
no	79.23 ± 14.08			55.39 ± 9.89			240.05 ± 40.32		

Note:

a- P < 0.05 when compared with stratum a;

b- P < 0.05 when compared with stratum b;

c- P < 0.05 when compared with stratum c;

d- P < 0.05 when compared with stratum d

**Table 4:** Assignment of independent variables in multifactor linear regression model

Independent variables	Independent variables
Age	Dummy variables are defined with reference to “≤21”, 22=(Z1 = 1, Z2 = 0, Z3 = 0);23=(Z1 = 0, Z2 = 1, Z3 = 0) ≥24=(Z1 = 0, Z2 = 0, Z3 = 1)
Gender	Female = 0;Male = 1
Educational background	Bachelor degree or above = 0;junior college or below = 1
Whether your parents support you becoming a nurse	No = 0;Yes = 1
Whether you choose the nursing profession voluntarily	No = 0;Yes = 1
Have you ever served as a student leader	No = 0;Yes = 1
Have you ever received a scholarship	No = 0;Yes = 1
Frequency of online theory courses since the COVID-19	Dummy variable for “≥11 times/week” as reference, ≤ 5 time/week=(Z1 = 1, Z2 = 0);6–10 time/week=(Z1 = 0, Z2 = 1)
Whether your willingness to engage in nursing has increased during the COVID-19 pandemic	No = 0;Yes = 1
Are you confident in clinical nursing practice	No = 0;Yes = 1
Professional identity	Original value
Emotional intelligence	Original value

**Table 5:** Results of multifactor linear regression model for work readiness

Variables	β	SE	t	P
Constant	54.852	9.980	5.496	< 0.001
**Age**				
≤ 21	0.0(reference)			
22	6.959	3.681	1.891	0.059
23	15.858	3.924	4.042	< 0.001
≥ 24	13.965	4.093	3.412	0.001
**Gender**				
Male	19.578	4.872	4.019	< 0.001
Female	0.0(reference)			
**Frequency of online theory courses since the COVID-19**				
≤ 5 times/week	−8.032	3.498	−2.296	0.022
6–10 times/week	−10.125	3.312	−3.057	0.002
≥ 11 times/week	0.0(reference)			
**Professional identity**	1.368	0.177	7.723	< 0.001
**Emotional intelligence**	1.340	0.142	9.442	< 0.001

Note: F = 424.386, P < 0.001;R^2^ = 0.5871, Adjusted R^2^ = 0.5797

**Table 6:** Results of the mediated effects model of work readiness

Effect	Coefficient	Bootstrap SE	95% Confidence Interval		P
			Lower	Upper	
**Direct effect**					
Emotional intelligence→Professional identity	0.805	0.024	0.755	0.850	< 0.001
Professional identity→Work readiness	0.562	0.086	0.395	0.729	< 0.001
Emotional intelligence→Work readiness	0.289	0.089	0.106	0.457	0.002
**Indirect effect**					
Emotional intelligence→Professional identity→Work readiness	0.452	0.073	0.319	0.603	< 0.001
**Total effect**					
Emotional intelligence→Professional identity	0.805	0.024	0.755	0.850	< 0.001
Professional identity→Work readiness	0.562	0.086	0.395	0.729	< 0.001
Emotional intelligence→Work readiness	0.741	0.033	0.668	0.800	0.001

## Data Availability

The datasets used during the study are available from the corresponding author on reasonable request.
